# The use of probiotics for improving lipid profiles in dyslipidemic individuals: an overview protocol

**DOI:** 10.1186/s13643-018-0826-2

**Published:** 2018-10-17

**Authors:** Patricia M. Fortes, Solomar M. Marques, Karolline A. Viana, Luciane R. Costa, Alessandra V. Naghettini, Paulo Sucasas Costa

**Affiliations:** 1 Health Sciences Graduate Program, Medicine School, Universidade Federal de Goiás (UFG), Rua 235, s/n, Setor Leste Universitário, Goiânia, GO CEP 74605-050 Brazil; 2 Department of Pediatrics, Hospital das Clinicas, UFG, Primeira Avenida, s/n, Setor Leste Universitário, Goiânia, GO CEP 74605-020 Brazil; 3 Dentistry Graduate Program, UFG, Primeira Avenida, s/n, esquina com Praça Universitária, Setor Leste Universitário, Goiânia, GO CEP 74605-220 Brazil; 4 Division of Pediatric Dentistry, Faculdade de Odontologia, UFG, Primeira Avenida, s/n, esquina com Praça Universitária, Setor Leste Universitário, Goiânia, GO CEP 74605-220 Brazil; 5 Health Education Graduate Program, Medicine School, UFG, Rua 235, s/n, Setor Leste Universitário, Goiânia, GO CEP 74605-050 Brazil

**Keywords:** Probiotic, Dyslipidemia, Hypercholesterolemia, Overview, Umbrella review

## Abstract

**Background:**

Dyslipidemia is a major risk factor in triggering cardiovascular events, which can lead to the death of millions of people around the world. Thus, several pharmacological and non-pharmacological therapeutic strategies have been developed in recent decades with the objective of improving lipid profiles, including the use of probiotics. Therefore, the purpose of this protocol is to describe the steps that will guide the construction of an overview to demonstrate the scientific evidence of the efficacy of probiotics in improving the lipid profile of dyslipidemic individuals and to propose specific recommendations regarding their use.

**Methods:**

The search will be conducted in the following databases: MEDLINE/PubMed, EMBASE, PROSPERO, Cochrane Library, CINAHL, JBI Database of Systematic Reviews and Implementation Reports, Google Scholar, and CADTH. Reviewers will select systematic evaluations and data analyses from randomized clinical trials that evaluated the effects of probiotics on lipid profiles. The studies will be analyzed for methodology quality by the AMSTAR 2 tool and risk of bias by ROBIS. The data will be extracted by three independent reviewers based on a data extraction sheet, which will include the most relevant variables for the analysis and interpretation of the results. The variables will be categorized and described in narrative form or in tables.

**Discussion:**

There are some systematic reviews about the use of probiotics to prevent and/or treat dyslipidemia; however, their outcomes related to the ability of probiotics to improve lipid profiles are conflicting. So, an overview on this topic is needed to clarify this important issue.

**Systematic review registration:**

PROSPERO CRD42017080328

**Electronic supplementary material:**

The online version of this article (10.1186/s13643-018-0826-2) contains supplementary material, which is available to authorized users.

## Background

Dyslipidemia is one of the most important cardiovascular risk factors (CRF) in triggering serious events. According to the World Health Organization (WHO), 17 million people died in 2015 from cardiovascular disease, accounting for about 31% of deaths worldwide in that period; acute myocardial infarction (AMI) and stroke were the main events diagnosed as a cause of death [1]. Therefore, dyslipidemia is still the most important modifiable risk factor associated with these events [2]. However, what makes controlling it challenging is that it has reached epidemic proportions, develops quietly, and is associated with other cardiovascular risk factors [3, 4].

Considering that the control of dyslipidemia is an important factor in the prevention of AMI and stroke, there are many protocols and guidelines with therapeutic regimens related to pharmacological and non-pharmacological treatments. Yet, controlling this condition has become increasingly difficult probably due to its multifactorial origin [5, 6]. Therefore, new treatment options have been proposed; among them, some scientific interest has focused on the use of probiotics, defined as “living microorganisms that in adequate quantities are able to bring benefits to host’s health” [7].

In recent decades, probiotics have been used in the management of different health conditions, demonstrating their benefits and safety when administered in humans, including dyslipidemic individuals [8]. The mechanisms of action by which different probiotic strains act in the control of dyslipidemia have been suggested from randomized clinical trials, highlighting (1) deconjugation and precipitation of bile acids by enzymatic action; (2) incorporation of cholesterol into the cell membrane of microorganisms; (3) inhibition of cholesterol transmembrane transporter expression in enterocytes; (4) cholesterol conversion to coprostanol; and (5) inhibition of hepatic synthesis of cholesterol [9].

Several studies, mainly clinical trials, have demonstrated the ability of some bacterial strains to reduce total cholesterol, low-density lipoprotein cholesterol (LDL-c), and triglycerides [10–15]. On the other hand, no changes were observed in the lipid profile after the use of probiotics in other studies [16–18]. In a preliminary literature search, we found systematic reviews (SRs) that generated contradictory results of probiotic use in dyslipidemic individuals [19–22]. Thus, an overview of SRs is required to support the healthcare decision-making on probiotics to treat dyslipidemia,

Therefore, the purpose of this protocol is to critically assess the scientific evidence presented in the systematic reviews currently available regarding the efficacy of probiotics in improving the lipid profile in dyslipidemic individuals. After this process, an overview will be drawn up showing the quality of such evidence that can enable health professionals to make specific recommendations for the use of probiotics in this context.

## Methods/design

The term “overview” refers to a review of systematic reviews and/or meta-analyses that compares studies and promotes a critical analysis of the main viable evidence about a particular issue, thus facilitating appropriate decision making [23]. The development of this protocol followed the guidelines presented by the Joanna Briggs Institute (JBI) for reviewers [23] and the recommendations proposed by PRISMA-P (Preferential Reporting Items for Systematic Review and Meta-analysis—Protocol) [24] (Additional file 1). The elaboration of the overview will follow the steps described in this protocol, and any deviation in the steps proposed here will be reported and justified. This overview protocol was registered in the PROSPERO database CRD42017080328.

### Research question

Does the use of probiotics improve the lipid profile in dyslipidemic individuals?

### Eligibility criteria

#### Types of study

Only systematic reviews which have been carried out from randomized clinical trials with or without meta-analyses, performed or not under Cochrane methodology will be included to allow a greater level of evidence [25]. Additionally, systematic reviews may have clearly demonstrated a research question that includes the acronym PICO (population, intervention, comparison/control, outcome(s)) [23, 26]. The SRs should have been published since January 2000, because this is the period of the publication of one of the first SR summarizing evidence about efficacy of probiotic use in the control of dyslipidemia [27]. Narrative reviews, protocols, reviews of animal studies, reviews of observational studies, and case series will not be included.

#### Participants

The participants included in SR should be well described regarding characteristics such as age, gender, sample size, and clinical conditions associated with dyslipidemia. Systematic reviews will be excluded if more than 80% of the participants in the sample are carriers of type 1 diabetes, hypothyroidism, nephrotic syndrome, or if they are pregnant women or alcoholics, because they might require other therapeutic interventions that would confound the probiotics effect.

#### Intervention

The probiotic should be the only intervention used to control dyslipidemia. Systematic reviews will be included if they are detailed in the study design: administration of treatment, presentation form, probiotic strain identification, dose and time of probiotic use, whether the groups were conducted in parallel or cross-over, and if there were associations with other factors that could interfere with the action of the probiotic (e.g., prebiotic, symbiotic, phytotherapeutic, neutracetic, soy, drugs).

#### Comparator

The selected SRs should demonstrate that the interventions were compared with a control group composed of placebo or non-therapeutic substance.

#### Outcomes

We will include SRs whose outcomes were related to changes in lipid profiles (total cholesterol, LDL cholesterol, HDL cholesterol, and triglycerides).

### Search strategy

#### Electronic databases

Two investigators (PMF and PSC) will carry out the searches using the following databases: PubMed–MEDLINE (www.ncbi.nlm.nih.gov/pubmed/), EMBASE (www.embase.com), Cochrane Library (www.cochranelibrary.com), PROSPERO (www.crd.york.ac.uk), CINAHL, and JBI Database of Systematic Reviews and Implementation Reports. All electronic search tools are available for free, with the exception of EMBASE. Databases for gray literature such as Google Scholar (www.scholar.google.com) and CADTH “Gray Matters Light” (http://www.cadth.ca//resources/finding-evidence/grey-matters) will be searched for non-indexed publications and government policies. The references cited in the selected studies will also be reviewed by manual search.

#### Search terms

**Fig. 1:**
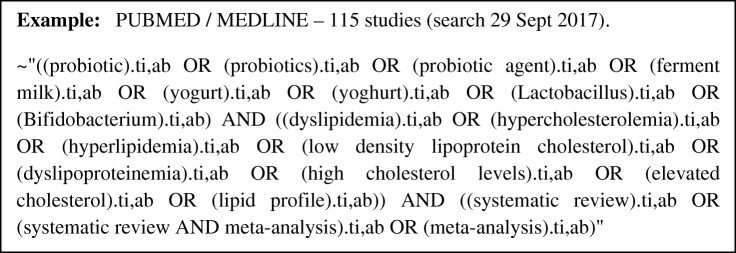
Combination of keywords and entry terms for search in the database

For the overview, authors used the key elements of the research question to define the descriptors selection. The purpose of this selection is to define the keywords that best described the eligibility criteria of the SRs to be selected without significantly narrowing the search. After identification, the terms will be used in combination, including keywords (MeSH–Medical Subject Headings) and entry terms that are present in the title, abstract, and full text (Additional file 2). The combinations of keywords and search strategy are exemplified in Fig. 1 and will be repeated for all databases.

### Selection of studies

Two independent reviewers (PMF and PSC) will select the studies. In case of disagreement between reviewers, a third reviewer will be consulted. Evaluation will begin with the exclusion of duplicate articles using the program Mendeley–Reference Management Software and Researcher Network, followed by application of the eligibility criteria (initially, the title and abstract). Selected articles from screening will undergo thorough reading for further examination of the eligibility criteria; eligible studies will be submitted for data extraction and quality assessment.

For a better visualization of the stages of study selection, a flow diagram will be set up as suggested by PRISMA (Preferential Reporting Items for Systematic Reviews and Meta-analyses) [28].

### Extraction of data

Each of the systematic reviews will be submitted to data extraction by three separate reviewers (PMF, SMM, and KAV), thus ensuring that important data will not be lost. The reviewers will use a standard form that will be initially tested in three studies; if necessary, it will be adapted. In order to adequately meet the interests research question of this overview, the following data will be extracted: (1) author, country, year of publication, number of RCTs included, funding (if any), language, and database (including gray literature); (2) characteristics of the population (sex, age, baseline diseases) and sample size; (3) intervention—strains of probiotics, study design, duration of treatment, dose, presentation form, association (prebiotic, symbiotic); (4) comparators (placebo or yogurt); (5) outcomes—changes in serum levels of total cholesterol, LDL-cholesterol, HDL-cholesterol, and triglycerides; (6) main adverse events related to probiotic supplementation (e.g., systemic—bacteremia, sepsis; and gastrointestinal—diarrhea, flatulence, abdominal pain); and (7) results—qualitative and quantitative (means, standard deviations, effect size, confidence interval, *p* value, and meta-analysis, if the data allow). If there are questions and disagreements between the reviewers in this process, a fourth reviewer will be consulted. If necessary, the RCTs analyzed in SRs will be checked for completion of some important data.

### **Assessment of the risk of bias and** methodological **quality**

The risk of bias will be evaluated using the ROBIS tool, according to specific guidelines for systematic reviews related to interventions. This tool is composed of three phases: phase 1—assessment of relevance (optional); phase 2—identification of concerns with the review process regarding the presence of bias, which is performed by four different domains and phase 3—judgment of the risk of bias through the summary of phase 2 findings. With the exception of phase 1, the other phases present answers that vary in (Y) “yes,” (PY) “probably yes,” (PN) “probably not,” (N) “no,” and (NI) “not informed” contained in each sub-area of the domains. The reason for concern is the risk of bias classified as high, low, or unclear [29].

The tool AMSTAR 2 (Additional file 3) (https://amstar.ca/docs/AMSTAR-2.pdf), which is composed of 16 domains, will be used in the evaluation of the methodological quality of the SRs. Of these 16 domains, 7 (2, 4, 7, 9, 11, 13, and 15) are considered critical for the reliability of SRs results, ranking the SR in high, moderate, low, and critically low [30]. For each domain, the answers will vary in “yes” (all criteria required by the domain have been checked), “partially yes” (only part of the criteria required in the domain have been checked), and “no” (the criteria required by the domain were not checked). Multiple flaws in non-critical domains (more than 3) will be considered capable of decreasing confidence in results; in this case, the classification will start from the moderate level. The domains will be analyzed independently, so that the response to a particular domain does not influence the evaluation of the others [30].

Both the bias risk assessment and the methodological quality evaluation of the reviews will be carried out by two independent reviewers (PMF, KAV). If disagreements occur, a third reviewer will be consulted (SMM).

### Data synthesis

Results of reviews will be summarized, and the characteristics of the variables will be described in narrative form or in tables where each one will be categorized and, if appropriate, allocated as a statistical measure. This will allow the comparison of studies, visualization of effects and critical interpretation of results.

A meta-analysis will not be performed, given that pooling the results of meta-analyses could introduce significant overlap and biased results, as a RCT could be included in more than one SR [31].

The calculation of the corrected covered area (CCA) will be applied to estimate the overlap of RCTs among the reviews included [31]. In order to calculate the CCA, a citation matrix containing the primary articles and the revisions will be elaborated and arranged in the order of publication of the revisions. If there is a high overlap of RCTs, the most recent revisions will be maintained and the impact of such findings on the evidence will be discussed.

The discordant results presented among SRs will be analyzed based on the algorithm proposed by Jadad et al. [32], which seeks to identify the likely causes of contradictory results in reviews that presented the same research question. Subsequently, it will be discussed how these different causes of disagreement between the results impact on the evidence.

## Discussion

The increase in the number of dyslipidemic individuals due to changes in life habits in all age groups has become a challenge for the control of this important cardiovascular risk factor [1]. Consequently, more and more investigations are carried out seeking therapeutic alternatives that can prevent the onset of cardiovascular events. Therefore, the purpose of this protocol is to present the search pathway for a critical analysis of current scientific evidence about the efficacy of probiotics use in the control of dyslipidemia. Furthermore, at the end of the execution of its stages, an overview can be produced to demonstrate the evidences found, contributing with greater clarity in the applicability of probiotics as a therapeutic resource against dyslipidemia, which may point out gaps and stimulate further research in this area.

## Additional files

Additional file 1: PRISMA-P (Preferred Reporting Items for Systematic review and Meta-Analysis Protocols) 2015 checklist. Recommended items to address in a systematic review protocol. (DOCX 41 kb)

Additional file 2: PubMed search. Combination of keywords and entry terms for search in the PUBMED (DOCX 14 kb)

Additional file 3: AMSTAR 2. Tool that will be used for critically appraising the systematic reviews included (PDF 250 kb)

## Abbreviations

AMI: Acute myocardial infraction
AMSTAR: A Measurement Tool to Assess Systematic Reviews
CA: Covered area
CCA: Corrected covered area
CRF: Cardiovascular risk factors
HDL-c: High-density lipoprotein cholesterol
JBI: Joanna Briggs Institute
LDL-c: Low-density lipoprotein cholesterol
MA: Meta-analyze
MeSH: Medical Subject Headings
PICO: Population, Intervention, Comparison, Outcome(s)
PRISMA: Preferential Reporting Items for Systematic Reviews and Meta-analyses
PRISMA-P: Preferred Reporting Items for Systematic Review and Meta-analysis–Protocol
RCT: Randomized controlled trials
SR: Systematic review
TIAB: Title/Abstract
WHO: World Health Organization
