# Supplementary material for: The use of probiotics for improving lipid profiles in dyslipidemic individuals: an overview protocol
Source: Syst Rev. 2018 Oct 17;7:165. doi: 10.1186/s13643-018-0826-2 (PMC6192191; doi:10.1186/s13643-018-0826-2)
Supplement: Supplementary file 2 — PubMed search. Combination of keywords and entry terms for search in the PUBMED (DOCX 14 kb) [file 13643_2018_826_MOESM2_ESM.docx]

**Additional file 3: Combination of keywords and entry terms for search in the PUBMED database.**

The search on PUBMED/MEDLINE electronic databases was performed on the 29^th^ September 2017 using the NICE Healthcare Databases Advanced Search (HDAS) interface.

1 (probiotic).ti,ab

2 (probiotics).ti,ab

3 (probiotic agent).ti,ab

4 (ferment milk).ti,ab

5 (yogurt).ti,ab

6 (yoghurt).ti,ab

7 (Lactobacillus).ti,ab

8 (Bifidobacterium).ti,ab

9 OR/(1-8)

10 (dyslipidemia).ti,ab

11 (hypercholesterolemia).ti,ab

12 (hyperlipidemia).ti,ab

13 (low density lipoprotein cholesterol).ti,ab

14 (dyslipoproteinemia).ti,ab

15 (high cholesterol levels).ti,ab

16 (elevated cholesterol).ti,ab

17 (lipid profile).ti,ab

18 OR/(10-17)

19 (systematic review).ti,ab

20 (systematic review AND meta-analysis).ti,ab

21 (meta-analysis).ti,ab

22 OR/(19-21)

23 (9 AND 18 AND 22)
